# Supplementary material for: The Systems Biology Research Tool: evolvable open-source software
Source: BMC Syst Biol. 2008 Jun 29;2:55. doi: 10.1186/1752-0509-2-55 (PMC2446383; doi:10.1186/1752-0509-2-55)
Supplement: Additional file 1 — SBRT Archive. An archive of the current version of the Systems Biology Research Tool. [file 1752-0509-2-55-S1.zip › sbrt-1.4.0/doc/developers_guide/api/sbrt/kernel/fba/IrrevRxnFluxomeV1.html]

IrrevRxnFluxomeV1


|  |  |  |
| --- | --- | --- |
| **Overview** \| **Package** \| **Class** \| **Use** \| **Tree** \| **Deprecated** \| **Index** \| **Help** | |  |
| **PREV CLASS**   **NEXT CLASS** | **FRAMES**    **NO FRAMES**     **All Classes** |  |
| SUMMARY: NESTED \| FIELD \| CONSTR \| METHOD | DETAIL: FIELD \| CONSTR \| METHOD |  |


---


## sbrt.kernel.fba Class IrrevRxnFluxomeV1

```
java.lang.Object
  sbrt.kernel.fba.IrrevRxnFluxomeV1
```

**All Implemented Interfaces:**: CatalyzedFluxome, Fluxome, IrrevRxnFluxome

---

``` public final class IrrevRxnFluxomeV1 extends java.lang.Object implements IrrevRxnFluxome ```

This class is a concrete implementation of IrrevRxnFluxome.
Instances of this class are immutable.

**Author:**
:   This class was written by Jeremiah Wright while in the Wagner lab.

---

| **Field Summary** | |
| --- | --- |
| `static Interval` | `OFF`             The constraint used by default for all source reactions: [0, 0]. |
| `static Interval` | `ON`             The constraint used by default for all internal and sink reactions: [0, Infinity). |


**Constructor Summary**

`IrrevRxnFluxomeV1(java.util.Map<java.lang.String,ChemRxn> rxns)`
  
          Constructs a new irreversible reaction fluxome from the
provided internal and exchange reactions.

`IrrevRxnFluxomeV1(java.util.Map<java.lang.String,ChemRxn> rxns,
java.util.Map<java.lang.String,Or<And<java.lang.String>>> catalysts)`
  
          Constructs a new irreversible reaction fluxome from the
provided internal and exchange reactions and catalysts.

`IrrevRxnFluxomeV1(java.util.Map<java.lang.String,IrrevRxn> internalRxns,
java.util.Map<java.lang.String,SourceRxn> sourceRxns,
java.util.Map<java.lang.String,SinkRxn> sinkRxns)`
  
          Constructs a new irreversible reaction fluxome from the
provided internal and exchange reactions.

`IrrevRxnFluxomeV1(java.util.Map<java.lang.String,IrrevRxn> internalRxns,
java.util.Map<java.lang.String,SourceRxn> sourceRxns,
java.util.Map<java.lang.String,SinkRxn> sinkRxns,
java.util.Map<java.lang.String,Or<And<java.lang.String>>> catalysts)`
  
          Constructs a new irreversible reaction fluxome from the
provided internal and exchange reactions and catalysts.


| **Method Summary** | |
| --- | --- |
| `int` | `addedConstraints()`             Returns the total number of constraints added to this fluxome. |
| `boolean` | `areReqCatalysts(java.util.Collection<java.lang.String> catalysts, java.lang.String rxnName)`             Indicates if the provided collection of catalysts is required for the reaction with the specified name. |
| `boolean` | `areRxnNames(java.util.Collection<java.lang.String> c)`             Indicates if every string in the provided collection is the name of a reaction contained in this fluxome. |
| `int` | `catalysts()`             Returns the total number of catalysts contained in this fluxome. |
| `void` | `checkCatalyst(java.lang.String catalyst)`             Ensures the provided string is a valid catalyst. |
| `void` | `checkRxnName(java.lang.String reactionName)`             Ensures the provided string is a valid reaction name. |
| `void` | `checkRxnNames(java.util.Collection<java.lang.String> rxnNames)`             Ensures each element of the provided collection is a valid reaction name. |
| `int` | `exchangeRxns()`             Returns the total number of exchange reactions contained in this fluxome. |
| `Or<And<java.lang.String>>` | `getCatalystRule(java.lang.String rxnName)`             Returns the boolean logic statement for the catalysts of the reaction with the provided name. |
| `java.util.Map<java.lang.String,Or<And<java.lang.String>>>` | `getCatalystRules()`             Returns all of the boolean logic statements regarding reaction catalysis. |
| `java.util.Set<java.lang.String>` | `getCatalysts()`             Returns the set of names of all catalysts contained in this fluxome. |
| `FluxConstraints` | `getConstraints()`             Returns all constraints in this fluxome. |
| `java.util.Collection<java.util.Set<java.lang.String>>` | `getEquivRxnNames()`             Returns a collection of sets of the names of stoichiometrically equivalent reactions contained in this fluxome. |
| `java.util.Set<java.lang.String>` | `getEquivRxnNames(java.lang.String reactionName)`             Returns the set of names of internal reactions in this fluxome that are stoichiometrically equivalent to the provided reaction. |
| `java.util.Map<java.lang.String,IrrevRxn>` | `getEquivRxns(java.lang.String rxnName)`             Returns the reactions in this fluxome that are stoichiometrically equivalent to the provided reaction. |
| `ExchangeRxn` | `getExchangeRxn(java.lang.String reactionName)`             Returns the exchange reaction in this fluxome with the provided name. |
| `java.util.Set<java.lang.String>` | `getExchangeRxnNames()`             Returns the set of names of all exchange reactions in this fluxome. |
| `java.util.Map<java.lang.String,ExchangeRxn>` | `getExchangeRxns()`             Returns all of the exchange reactions contained in this fluxome. |
| `Interval` | `getFluxConstraint(java.lang.String reactionName)`             Returns the flux constraint for the provided reaction. |
| `java.util.Map<java.lang.String,Interval>` | `getFluxConstraints()`             Returns the constraints on each flux in this fluxome. |
| `IrrevRxn` | `getInternalRxn(java.lang.String reactionName)`             Returns the internal reaction in this fluxome with the provided name. |
| `java.util.Set<java.lang.String>` | `getInternalRxnNames()`             Returns the set of names of all internal reactions in this fluxome. |
| `java.util.Map<java.lang.String,IrrevRxn>` | `getInternalRxns()`             Returns all of the internal reactions in this fluxome. |
| `java.util.Map<java.lang.String,IrrevRxn>` | `getInternalRxns(java.util.Collection<java.lang.String> rxnNames)`             Returns the internal reactions whose names appear in the provided collection of reaction names. |
| `java.util.Set<java.lang.String>` | `getReverseRxnNames(java.lang.String reactionName)`             Returns the names of all of the reactions in this fluxome that are stoichiometrically equivalent to the provided reaction in reverse. |
| `java.util.Map<java.lang.String,ChemRxn>` | `getReverseRxns(java.lang.String reactionName)`             Returns all of the reactions in this fluxome that are stoichiometrically equivalent to the provided reaction in reverse. |
| `ChemRxn` | `getRxn(java.lang.String reactionName)`             Returns the chemical reaction in this fluxome with the provided name. |
| `Interval` | `getRxnExprConstraint(MathExpr<java.lang.String> rxnNameExpr)`             Returns the constraint associated with the expression provided. |
| `java.util.Set<java.lang.String>` | `getRxnNames()`             Returns the set of names of all chemical reactions contained in this fluxome. |
| `java.util.Set<java.lang.String>` | `getRxnNames(java.lang.String catalyst)`             Returns the names of the reactions catalyzed by the provided catalyst. |
| `java.util.Map<java.lang.String,ChemRxn>` | `getRxns()`             Returns all chemical reactions contained in this fluxome. |
| `java.util.Map<java.lang.String,ChemRxn>` | `getRxns(java.util.Collection<java.lang.String> rxnNames)`             Returns the chemical reactions with the provided names. |
| `java.util.Map<java.lang.String,IrrevRxn>` | `getRxns(java.lang.String catalyst)`             Returns all of the internal reactions contained in this fluxome that are catalyzed by the provided catalyst. |
| `SinkRxn` | `getSinkRxn(java.lang.String reactionName)`             Returns the sink reaction in this fluxome with the provided name. |
| `java.util.Set<java.lang.String>` | `getSinkRxnNames()`             Returns the set of names of all sinks in this fluxome. |
| `java.util.Map<java.lang.String,SinkRxn>` | `getSinkRxns()`             Returns all of the sinks in this fluxome. |
| `java.util.Map<java.lang.String,SinkRxn>` | `getSinkRxns(java.util.Collection<java.lang.String> rxnNames)`             Returns the sink reactions whose names appear in the provided collection of reaction names. |
| `SourceRxn` | `getSourceRxn(java.lang.String reactionName)`             Returns the source reaction in this fluxome with the provided name. |
| `java.util.Set<java.lang.String>` | `getSourceRxnNames()`             Returns the set of names of all sources in this fluxome. |
| `java.util.Map<java.lang.String,SourceRxn>` | `getSourceRxns()`             Returns all of the sources in this fluxome. |
| `java.util.Map<java.lang.String,SourceRxn>` | `getSourceRxns(java.util.Collection<java.lang.String> rxnNames)`             Returns the source reactions whose names appear in the provided collection of reaction names. |
| `java.util.Set<java.lang.String>` | `getSpecies()`             Returns the set of names of all chemical species participating in reactions contained in this fluxome. |
| `SparseDoubleMatrix<java.lang.String,java.lang.String>` | `getStoichiometryMatrix()`             Returns the stoichiometry matrix of this fluxome. |
| `int` | `internalRxns()`             Returns the total number of internal reactions contained in this fluxome. |
| `boolean` | `isCatalyst(java.lang.String s)`             Indicates if the provided string is the name of an catalyst contained in this fluxome. |
| `boolean` | `isFluxVector(java.util.Map<java.lang.String,java.lang.Double> fluxValues, double tolerance)`             Indicates if the provided flux vector lies within the feasible space of this fluxome, given the provided tolerance value. |
| `boolean` | `isReqCatalyst(java.lang.String catalyst, java.lang.String rxnName)`             Indicates if the specified catalyst is required for the reaction with the specified name. |
| `boolean` | `isRxnName(java.lang.String s)`             Indicates if the provided string is the name of a reaction contained in this fluxome. |
| `boolean` | `isSpecies(java.lang.String s)`             Indicates if the provided string is the name of a chemical species contained in this fluxome. |
| `boolean` | `isValid(Interval c, java.lang.String rxnName, double tolerance)`             Indicates if the provided interval is a valid flux constraint for the reaction having the provided name. |
| `static IrrevRxnFluxomeV1` | `make(java.util.Collection<java.lang.String> reactionNames, IrrevRxnFluxome fluxome)`             Returns an irreversible reaction fluxome composed of the reactions in the provided fluxome with the names in the provided collection. |
| `int` | `redundantRxns()`             Returns the total number of redundant reactions contained in this fluxome. |
| `IrrevRxnFluxomeV1` | `replaceConstraints(FluxConstraints fluxConstraints, double constraintTolerance)`             Returns a copy of this fluxome, but with all constraints replaced by those provided. |
| `int` | `rxns()`             Returns the total number of chemical reactions contained in this fluxome. |
| `int` | `sinkRxns()`             Returns the total number of sink reactions contained in this fluxome. |
| `int` | `sourceRxns()`             Returns the total number of source reactions contained in this fluxome. |
| `int` | `species()`             Returns the total number of chemical species contained in this fluxome. |

**Methods inherited from class java.lang.Object**

`clone, equals, finalize, getClass, hashCode, notify, notifyAll, toString, wait, wait, wait`

**Field Detail**

### ON

```
public static final Interval ON
```

:   The constraint used by default for all internal and
    sink reactions: [0, Infinity).

---


### OFF

```
public static final Interval OFF
```

:   The constraint used by default for all
    source reactions: [0, 0].


**Constructor Detail**

### IrrevRxnFluxomeV1

```
public IrrevRxnFluxomeV1(java.util.Map<java.lang.String,IrrevRxn> internalRxns,
                         java.util.Map<java.lang.String,SourceRxn> sourceRxns,
                         java.util.Map<java.lang.String,SinkRxn> sinkRxns,
                         java.util.Map<java.lang.String,Or<And<java.lang.String>>> catalysts)
```

:   Constructs a new irreversible reaction fluxome from the
    provided internal and exchange reactions and catalysts. The flux of each
    reaction is constrained to its default value.

    **Parameters:**: `internalRxns` - reaction names mapped to the internal reactions to be contained in this fluxome.: `sourceRxns` - reaction names mapped to the sources to be contained in this fluxome.: `sinkRxns` - reaction names mapped to the sinks to be contained in this fluxome.: `catalysts` - the names of internal reactions mapped to the boolean logic rules governing their catalysis. Only the names of catalyzed reactions need to be present as keys in this map.

---


### IrrevRxnFluxomeV1

```
public IrrevRxnFluxomeV1(java.util.Map<java.lang.String,IrrevRxn> internalRxns,
                         java.util.Map<java.lang.String,SourceRxn> sourceRxns,
                         java.util.Map<java.lang.String,SinkRxn> sinkRxns)
```

:   Constructs a new irreversible reaction fluxome from the
    provided internal and exchange reactions. The flux of each
    reaction is constrained to its default value.

    **Parameters:**: `internalRxns` - reaction names mapped to the internal reactions to be contained in this fluxome.: `sourceRxns` - reaction names mapped to the sources to be contained in this fluxome.: `sinkRxns` - reaction names mapped to the sinks to be contained in this fluxome.

---


### IrrevRxnFluxomeV1

```
public IrrevRxnFluxomeV1(java.util.Map<java.lang.String,ChemRxn> rxns,
                         java.util.Map<java.lang.String,Or<And<java.lang.String>>> catalysts)
```

:   Constructs a new irreversible reaction fluxome from the
    provided internal and exchange reactions and catalysts. The flux of each
    reaction is constrained to its default value.

    **Parameters:**: `rxns` - reaction names mapped to the internal and exchange reactions to be contained in this fluxome.: `catalysts` - the names of internal reactions mapped to the boolean logic rules governing their catalysis. Only the names of catalyzed reactions need to be present as keys in this map.

---


### IrrevRxnFluxomeV1

```
public IrrevRxnFluxomeV1(java.util.Map<java.lang.String,ChemRxn> rxns)
```

:   Constructs a new irreversible reaction fluxome from the
    provided internal and exchange reactions. The flux of each
    reaction is constrained to its default value.

    **Parameters:**: `rxns` - reaction names mapped to the internal and exchange reactions to be contained in this fluxome.


**Method Detail**

### checkRxnNames

```
public void checkRxnNames(java.util.Collection<java.lang.String> rxnNames)
```

:   Ensures each element of the provided collection is a valid reaction name.

    :   **Specified by:**: `checkRxnNames` in interface `IrrevRxnFluxome`
    :   **Parameters:**: `rxnNames` - a collection of strings. **Throws:**: `java.lang.IllegalArgumentException` - if any element of this collection is not a valid reaction name.

---


### checkRxnName

```
public void checkRxnName(java.lang.String reactionName)
```

:   Ensures the provided string is a valid reaction name.

    :   **Specified by:**: `checkRxnName` in interface `IrrevRxnFluxome`
    :   **Parameters:**: `reactionName` - a potential reaction name. **Throws:**: `java.lang.IllegalArgumentException` - if the provided string is not a valid reaction name.

---


### checkCatalyst

```
public void checkCatalyst(java.lang.String catalyst)
```

:   Ensures the provided string is a valid catalyst.

    :   **Parameters:**: `catalyst` - a potential catalyst. **Throws:**: `java.lang.IllegalArgumentException` - if the provided string is not a valid catalyst.

---


### replaceConstraints

```
public IrrevRxnFluxomeV1 replaceConstraints(FluxConstraints fluxConstraints,
                                            double constraintTolerance)
```

:   Returns a copy of this fluxome, but with all constraints
    replaced by those provided.

    :   **Specified by:**: `replaceConstraints` in interface `CatalyzedFluxome` **Specified by:**: `replaceConstraints` in interface `Fluxome` **Specified by:**: `replaceConstraints` in interface `IrrevRxnFluxome`
    :   **Parameters:**: `fluxConstraints` - the new set of constraints on the system.: `constraintTolerance` - the amount by which a lower or upper bound from a provided constraint can violate the constraint `[0, Infinity)`. **Returns:**: a copy of this fluxome, but with all constraints replaced by those provided.

---


### getConstraints

```
public FluxConstraints getConstraints()
```

:   Returns all constraints in this fluxome.

    :   **Specified by:**: `getConstraints` in interface `Fluxome`
    :   **Returns:**: all constraints in this fluxome.

---


### getFluxConstraints

```
public java.util.Map<java.lang.String,Interval> getFluxConstraints()
```

:   Returns the constraints on each flux in this fluxome.

    :   **Returns:**: each reaction name in this fluxome mapped to its flux constraint.

---


### getFluxConstraint

```
public Interval getFluxConstraint(java.lang.String reactionName)
```

:   Returns the flux constraint for the provided reaction.

    :   **Specified by:**: `getFluxConstraint` in interface `Fluxome`
    :   **Parameters:**: `reactionName` - the name of the reaction whose flux constraint will be returned. **Returns:**: the flux constraint for the provided reaction.

---


### getRxnExprConstraint

```
public Interval getRxnExprConstraint(MathExpr<java.lang.String> rxnNameExpr)
```

:   Returns the constraint associated with the expression provided. If the
    expression provided does not exist in this fluxome, the constraint
    (-Infinity, Infinity) is returned.

    :   **Specified by:**: `getRxnExprConstraint` in interface `Fluxome`
    :   **Parameters:**: `rxnNameExpr` - the mathematical expression of reaction names whose constraint is to be returned. **Returns:**: the constraint on the provided mathematical expression of fluxes.

---


### getExchangeRxns

```
public java.util.Map<java.lang.String,ExchangeRxn> getExchangeRxns()
```

:   Returns all of the exchange reactions contained in this fluxome.
    This is the union of all sources and sinks.

    :   **Specified by:**: `getExchangeRxns` in interface `Fluxome`
    :   **Returns:**: the names of all exchange reactions in this fluxome mapped to their corresponding reaction. **See Also:**: `getSourceRxns()`, `getSinkRxns()`

---


### getRxns

```
public java.util.Map<java.lang.String,IrrevRxn> getRxns(java.lang.String catalyst)
```

:   Returns all of the internal reactions contained in this fluxome
    that are catalyzed by the provided catalyst.

    :   **Specified by:**: `getRxns` in interface `CatalyzedFluxome`
    :   **Parameters:**: `catalyst` - the name of the catalyst whose reactions are to be returned. **Returns:**: the set of all internal reactions contained in this fluxome that are catalyzed by the provided catalyst. **Throws:**: `java.lang.IllegalArgumentException` - if the provided catalyst does not exist in this fluxome.

---


### getRxns

```
public java.util.Map<java.lang.String,ChemRxn> getRxns()
```

:   Returns all chemical reactions contained in this fluxome.
    This is the union of all internal and exchange reactions.

    :   **Specified by:**: `getRxns` in interface `Fluxome`
    :   **Returns:**: the names of all reactions contained in this fluxome mapped to their corresponding reaction. **See Also:**: `getInternalRxns()`, `getSourceRxns()`, `getSinkRxns()`

---


### getRxns

```
public java.util.Map<java.lang.String,ChemRxn> getRxns(java.util.Collection<java.lang.String> rxnNames)
```

:   Returns the chemical reactions with the provided names.

    :   **Specified by:**: `getRxns` in interface `Fluxome`
    :   **Parameters:**: `rxnNames` - the names of the reactions to be returned. **Returns:**: the provided reaction names mapped to their corresponding reaction. **Throws:**: `java.lang.IllegalArgumentException` - if any of the provided names are invalid.

---


### getRxn

```
public ChemRxn getRxn(java.lang.String reactionName)
```

:   Returns the chemical reaction in this fluxome with the provided name.

    :   **Specified by:**: `getRxn` in interface `Fluxome`
    :   **Parameters:**: `reactionName` - the name of the chemical reaction to be returned. **Returns:**: the chemical reaction in this fluxome with the provided name. **Throws:**: `java.lang.IllegalArgumentException` - if the provided string is not a valid reaction name. **See Also:**: `getInternalRxn(String)`, `getSourceRxn(String)`, `getSinkRxn(String)`

---


### getExchangeRxn

```
public ExchangeRxn getExchangeRxn(java.lang.String reactionName)
```

:   Returns the exchange reaction in this fluxome with the provided name.

    :   **Parameters:**: `reactionName` - the name of the exchange reaction to be returned. **Returns:**: the chemical reaction in this fluxome with the provided name. **Throws:**: `java.lang.IllegalArgumentException` - if the provided string is not the name of an exchange reaction in this fluxome. **See Also:**: `getSourceRxn(String)`, `getSinkRxn(String)`

---


### getInternalRxn

```
public IrrevRxn getInternalRxn(java.lang.String reactionName)
```

:   Returns the internal reaction in this fluxome with the provided name.

    :   **Specified by:**: `getInternalRxn` in interface `IrrevRxnFluxome`
    :   **Parameters:**: `reactionName` - the name of the internal reaction to be returned. **Returns:**: the internal reaction in this fluxome with the provided name. **Throws:**: `java.lang.IllegalArgumentException` - if the provided string is not the name of an internal reaction in this fluxome.

---


### getRxnNames

```
public java.util.Set<java.lang.String> getRxnNames(java.lang.String catalyst)
```

:   Returns the names of the reactions catalyzed by the provided catalyst.

    :   **Specified by:**: `getRxnNames` in interface `CatalyzedFluxome`
    :   **Parameters:**: `catalyst` - the name of a catalyst in this fluxome. **Returns:**: a set of names of the reactions catalyzed by the provided catalyst. **Throws:**: `java.lang.IllegalArgumentException` - if the provided string is not the name of a catalyst in this fluxome.

---


### getRxnNames

```
public java.util.Set<java.lang.String> getRxnNames()
```

:   Returns the set of names of all chemical reactions
    contained in this fluxome. This set is the union
    of the names of internal and exchange reactions.

    :   **Specified by:**: `getRxnNames` in interface `Fluxome`
    :   **Returns:**: the set of names of all chemical reactions contained in this fluxome. **See Also:**: `getInternalRxnNames()`, `getSinkRxnNames()`, `getSourceRxnNames()`

---


### getInternalRxnNames

```
public java.util.Set<java.lang.String> getInternalRxnNames()
```

:   Returns the set of names of all internal reactions in this fluxome.

    :   **Specified by:**: `getInternalRxnNames` in interface `Fluxome`
    :   **Returns:**: the set of names of all internal reactions in this fluxome.

---


### getInternalRxns

```
public java.util.Map<java.lang.String,IrrevRxn> getInternalRxns()
```

:   Returns all of the internal reactions in this fluxome.

    :   **Specified by:**: `getInternalRxns` in interface `Fluxome` **Specified by:**: `getInternalRxns` in interface `IrrevRxnFluxome`
    :   **Returns:**: all of the internal reactions in this fluxome.

---


### getExchangeRxnNames

```
public java.util.Set<java.lang.String> getExchangeRxnNames()
```

:   Returns the set of names of all exchange reactions in this fluxome.

    :   **Specified by:**: `getExchangeRxnNames` in interface `Fluxome`
    :   **Returns:**: the set of names of all exchange reactions in this fluxome. **See Also:**: `getSourceRxnNames()`, `getSinkRxnNames()`

---


### getSourceRxnNames

```
public java.util.Set<java.lang.String> getSourceRxnNames()
```

:   Returns the set of names of all sources in this fluxome.

    :   **Specified by:**: `getSourceRxnNames` in interface `IrrevRxnFluxome`
    :   **Returns:**: the set of names of all sources in this fluxome.

---


### getSinkRxnNames

```
public java.util.Set<java.lang.String> getSinkRxnNames()
```

:   Returns the set of names of all sinks in this fluxome.

    :   **Specified by:**: `getSinkRxnNames` in interface `IrrevRxnFluxome`
    :   **Returns:**: the set of names of all sinks in this fluxome.

---


### getSourceRxns

```
public java.util.Map<java.lang.String,SourceRxn> getSourceRxns()
```

:   Returns all of the sources in this fluxome.

    :   **Specified by:**: `getSourceRxns` in interface `IrrevRxnFluxome`
    :   **Returns:**: the name of all of the sources in this fluxome mapped to their corresponding reaction.

---


### getSinkRxns

```
public java.util.Map<java.lang.String,SinkRxn> getSinkRxns()
```

:   Returns all of the sinks in this fluxome.

    :   **Specified by:**: `getSinkRxns` in interface `IrrevRxnFluxome`
    :   **Returns:**: the name of all of the sinks in this fluxome mapped to their corresponding reaction.

---


### getSourceRxn

```
public SourceRxn getSourceRxn(java.lang.String reactionName)
```

:   Returns the source reaction in this fluxome with the provided name.

    :   **Specified by:**: `getSourceRxn` in interface `IrrevRxnFluxome`
    :   **Parameters:**: `reactionName` - the name of the source reaction to be returned. **Returns:**: the source reaction in this fluxome with the provided name. **Throws:**: `java.lang.IllegalArgumentException` - if the provided string is not the name of a source reaction in this fluxome.

---


### getSinkRxn

```
public SinkRxn getSinkRxn(java.lang.String reactionName)
```

:   Returns the sink reaction in this fluxome with the provided name.

    :   **Specified by:**: `getSinkRxn` in interface `IrrevRxnFluxome`
    :   **Parameters:**: `reactionName` - the name of the sink reaction to be returned. **Returns:**: the sink reaction in this fluxome with the provided name. **Throws:**: `java.lang.IllegalArgumentException` - if the provided string is not the name of a sink reaction in this fluxome.

---


### getEquivRxnNames

```
public java.util.Collection<java.util.Set<java.lang.String>> getEquivRxnNames()
```

:   Returns a collection of sets of the names of stoichiometrically
    equivalent reactions contained in this fluxome.

    :   **Specified by:**: `getEquivRxnNames` in interface `Fluxome`
    :   **Returns:**: a collection of sets of the names of stoichiometrically equivalent reactions contained in this fluxome.

---


### getEquivRxns

```
public java.util.Map<java.lang.String,IrrevRxn> getEquivRxns(java.lang.String rxnName)
```

:   Returns the reactions in this fluxome that are
    stoichiometrically equivalent to the provided reaction.

    :   **Specified by:**: `getEquivRxns` in interface `IrrevRxnFluxome`
    :   **Parameters:**: `rxnName` - the name of the internal reaction whose stoichiometrically equivalent reactions will be returned. **Returns:**: the names of internal reactions in this fluxome that are stoichiometrically equivalent to the provided reaction mapped to their corresponding reaction. **Throws:**: `java.lang.IllegalArgumentException` - if the provided string is not the name of an internal reaction.

---


### getEquivRxnNames

```
public java.util.Set<java.lang.String> getEquivRxnNames(java.lang.String reactionName)
```

:   Returns the set of names of internal reactions in this fluxome that are
    stoichiometrically equivalent to the provided reaction.

    :   **Specified by:**: `getEquivRxnNames` in interface `Fluxome`
    :   **Parameters:**: `reactionName` - the name of the reaction whose stoichiometrically equivalent reactions will be returned. **Returns:**: the set of names of reactions in this fluxome that are stoichiometrically equivalent to the provided reaction. **Throws:**: `java.lang.IllegalArgumentException` - if the provided string is not the name of an internal reaction.

---


### getReverseRxnNames

```
public java.util.Set<java.lang.String> getReverseRxnNames(java.lang.String reactionName)
```

:   Returns the names of all of the reactions in this fluxome that are
    stoichiometrically equivalent to the provided reaction in reverse.
    Source and sink reactions are included in this search.

    :   **Specified by:**: `getReverseRxnNames` in interface `Fluxome`
    :   **Parameters:**: `reactionName` - the name of the reaction for which the stoichiometrically equivalent reverse reaction names will be returned. **Returns:**: the names of all reactions in this fluxome that are stoichiometrically equivalent to the provided reaction in reverse.

---


### getReverseRxns

```
public java.util.Map<java.lang.String,ChemRxn> getReverseRxns(java.lang.String reactionName)
```

:   Returns all of the reactions in this fluxome that are
    stoichiometrically equivalent to the provided reaction in reverse.
    Source and sink reactions are included in this search.

    :   **Specified by:**: `getReverseRxns` in interface `IrrevRxnFluxome`
    :   **Parameters:**: `reactionName` - the name of the reaction for which the stoichiometrically equivalent reverse reactions will be returned. **Returns:**: the names of all reactions in this fluxome that are stoichiometrically equivalent to the provided reaction in reverse mapped to their corresponding internal, source, or sink reaction.

---


### rxns

```
public int rxns()
```

:   Returns the total number of chemical reactions contained
    in this fluxome.

    :   **Specified by:**: `rxns` in interface `Fluxome`
    :   **Returns:**: the total number of chemical reactions contained in this fluxome.

---


### internalRxns

```
public int internalRxns()
```

:   Returns the total number of internal reactions contained
    in this fluxome.

    :   **Specified by:**: `internalRxns` in interface `Fluxome`
    :   **Returns:**: the total number of internal reactions contained in this fluxome.

---


### exchangeRxns

```
public int exchangeRxns()
```

:   Returns the total number of exchange reactions contained
    in this fluxome.

    :   **Specified by:**: `exchangeRxns` in interface `Fluxome`
    :   **Returns:**: the total number of exchange reactions contained in this fluxome.

---


### sourceRxns

```
public int sourceRxns()
```

:   Returns the total number of source reactions contained
    in this fluxome.

    :   **Specified by:**: `sourceRxns` in interface `IrrevRxnFluxome`
    :   **Returns:**: the total number of source reactions contained in this fluxome.

---


### sinkRxns

```
public int sinkRxns()
```

:   Returns the total number of sink reactions contained
    in this fluxome.

    :   **Specified by:**: `sinkRxns` in interface `IrrevRxnFluxome`
    :   **Returns:**: the total number of sink reactions contained in this fluxome.

---


### addedConstraints

```
public int addedConstraints()
```

:   Returns the total number of constraints added to this fluxome.

    :   **Specified by:**: `addedConstraints` in interface `Fluxome`
    :   **Returns:**: the total number of constraints added to this fluxome. **See Also:**: `replaceConstraints(FluxConstraints, double)`

---


### catalysts

```
public int catalysts()
```

:   Returns the total number of catalysts contained in this fluxome.

    :   **Specified by:**: `catalysts` in interface `CatalyzedFluxome`
    :   **Returns:**: the total number of catalysts contained in this fluxome.

---


### species

```
public int species()
```

:   Returns the total number of chemical species contained in this fluxome.

    :   **Specified by:**: `species` in interface `Fluxome`
    :   **Returns:**: the total number of chemical species contained in this fluxome.

---


### redundantRxns

```
public int redundantRxns()
```

:   Returns the total number of redundant reactions contained in this
    fluxome.

    For any group of stoichiometrically reactions of size N, the number of
    redundant reactions within that group is N - 1. The total number of
    redundant reactions in a fluxome equals the sum of redundant reactions
    over each group of stoichiometrically equivalent reactions.

    :   **Specified by:**: `redundantRxns` in interface `Fluxome`
    :   **Returns:**: the total number of redundant reactions contained in this fluxome. **See Also:**: `getEquivRxnNames()`

---


### getCatalysts

```
public java.util.Set<java.lang.String> getCatalysts()
```

:   Returns the set of names of all catalysts contained in this fluxome.

    :   **Specified by:**: `getCatalysts` in interface `CatalyzedFluxome`
    :   **Returns:**: the set of names of all catalysts contained in this fluxome.

---


### getSpecies

```
public java.util.Set<java.lang.String> getSpecies()
```

:   Returns the set of names of all chemical species participating
    in reactions contained in this fluxome.

    :   **Specified by:**: `getSpecies` in interface `Fluxome`
    :   **Returns:**: the set of names of all chemical species participating in reactions contained in this fluxome.

---


### isSpecies

```
public boolean isSpecies(java.lang.String s)
```

:   Indicates if the provided string is the name of a chemical species
    contained in this fluxome.

    :   **Specified by:**: `isSpecies` in interface `Fluxome`
    :   **Parameters:**: `s` - the string to test. **Returns:**: `true` if the string is the name of a chemical species contained in this fluxome; `false` otherwise.

---


### isCatalyst

```
public boolean isCatalyst(java.lang.String s)
```

:   Indicates if the provided string is the name of an catalyst contained in
    this fluxome.

    :   **Specified by:**: `isCatalyst` in interface `CatalyzedFluxome`
    :   **Parameters:**: `s` - the string to test. **Returns:**: `true` if the string is the name of a catalyst contained in this fluxome; `false` otherwise.

---


### isRxnName

```
public boolean isRxnName(java.lang.String s)
```

:   Indicates if the provided string is the name of a reaction contained in
    this fluxome.

    :   **Specified by:**: `isRxnName` in interface `Fluxome`
    :   **Parameters:**: `s` - the string to test. **Returns:**: `true` if the string is the name of a reaction contained in this fluxome; `false` otherwise.

---


### areRxnNames

```
public boolean areRxnNames(java.util.Collection<java.lang.String> c)
```

:   Indicates if every string in the provided collection is the name
    of a reaction contained in this fluxome.

    :   **Specified by:**: `areRxnNames` in interface `Fluxome`
    :   **Parameters:**: `c` - a collection of strings. **Returns:**: `true` if every string is a valid reaction name; `false` otherwise.

---


### isValid

```
public boolean isValid(Interval c,
                       java.lang.String rxnName,
                       double tolerance)
```

:   Indicates if the provided interval is a valid
    flux constraint for the reaction having the provided
    name.

    :   **Specified by:**: `isValid` in interface `Fluxome`
    :   **Parameters:**: `c` - the flux constraint to test.: `rxnName` - the name of the reaction.: `tolerance` - the numerical tolerance. **Returns:**: `c.getLower() >= -1 * tolerance` **Throws:**: `java.lang.IllegalArgumentException` - if the provided reaction name does not exist in this fluxome; or if the provided tolerance is < 0, NaN, or infinite.: `java.lang.NullPointerException` - if the provided constraint is `null`

---


### getStoichiometryMatrix

```
public SparseDoubleMatrix<java.lang.String,java.lang.String> getStoichiometryMatrix()
```

:   Returns the stoichiometry matrix of this fluxome.

    :   **Specified by:**: `getStoichiometryMatrix` in interface `Fluxome`
    :   **Returns:**: the stoichiometry matrix of this fluxome. Row indices in this matrix are the names of chemical species, and column indices are reaction names.

---


### isFluxVector

```
public boolean isFluxVector(java.util.Map<java.lang.String,java.lang.Double> fluxValues,
                            double tolerance)
```

:   Indicates if the provided flux vector lies within the feasible space
    of this fluxome, given the provided tolerance value.

    :   **Specified by:**: `isFluxVector` in interface `Fluxome`
    :   **Parameters:**: `fluxValues` - reaction names mapped to flux values.: `tolerance` - the numerical tolerance. **Returns:**: `true` if the vector satisfies all constraints; `false` otherwise.

---


### make

```
public static IrrevRxnFluxomeV1 make(java.util.Collection<java.lang.String> reactionNames,
                                     IrrevRxnFluxome fluxome)
```

:   Returns an irreversible reaction fluxome composed of the reactions in the
    provided fluxome with the names in the provided collection. This method
    can be used to copy an entire fluxome or to create a sub-fluxome. The
    constraints in the returned fluxome are set to their default values, that
    is, they are not copied from the provided fluxome.

    :   **Parameters:**: `reactionNames` - the names of reactions in the provided fluxome used to compose the returned fluxome.: `fluxome` - the fluxome used to compose the returned fluxome. **Returns:**: an irreversible reaction fluxome composed of the reactions in the provided fluxome with the names in the provided collection.

---


### getCatalystRules

```
public java.util.Map<java.lang.String,Or<And<java.lang.String>>> getCatalystRules()
```

:   Returns all of the boolean logic statements regarding reaction catalysis.

    :   **Specified by:**: `getCatalystRules` in interface `CatalyzedFluxome` **Specified by:**: `getCatalystRules` in interface `IrrevRxnFluxome`
    :   **Returns:**: the names of catalyzed reactions mapped to their boolean logic rules of their catalyst(s).

---


### getCatalystRule

```
public Or<And<java.lang.String>> getCatalystRule(java.lang.String rxnName)
```

:   Returns the boolean logic statement for the catalysts of the reaction
    with the provided name.

    :   **Specified by:**: `getCatalystRule` in interface `CatalyzedFluxome` **Specified by:**: `getCatalystRule` in interface `IrrevRxnFluxome`
    :   **Parameters:**: `rxnName` - the name of the reaction for which the catalyst rules are to be returned. **Returns:**: the boolean logic statement for the catalysts of the reaction with the provided name. If the specified reaction is not catalyzed, an empty (non-null) rule is returned.

---


### getInternalRxns

```
public java.util.Map<java.lang.String,IrrevRxn> getInternalRxns(java.util.Collection<java.lang.String> rxnNames)
```

:   Returns the internal reactions whose names appear in the provided
    collection of reaction names. Every string in the provided collection
    must be the name of a reaction in this fluxome, but the names of exchange
    reactions are ignored.

    :   **Specified by:**: `getInternalRxns` in interface `IrrevRxnFluxome`
    :   **Parameters:**: `rxnNames` - a collection of reaction names. **Returns:**: the names of internal reactions contained in `rxnNames` mapped to their corresponding reaction. **Throws:**: `java.lang.IllegalArgumentException` - if a string in `rxnNames` is not the name of a reaction in this fluxome.

---


### getSinkRxns

```
public java.util.Map<java.lang.String,SinkRxn> getSinkRxns(java.util.Collection<java.lang.String> rxnNames)
```

:   Returns the sink reactions whose names appear in the provided
    collection of reaction names. Every string in the provided collection
    must be the name of a reaction in this fluxome, but the names of internal
    and source reactions are ignored.

    :   **Specified by:**: `getSinkRxns` in interface `IrrevRxnFluxome`
    :   **Parameters:**: `rxnNames` - a collection of reaction names. **Returns:**: the names of sink reactions contained in `rxnNames` mapped to their corresponding reaction. **Throws:**: `java.lang.IllegalArgumentException` - if a string in `rxnNames` is not the name of a reaction in this fluxome.

---


### getSourceRxns

```
public java.util.Map<java.lang.String,SourceRxn> getSourceRxns(java.util.Collection<java.lang.String> rxnNames)
```

:   Returns the source reactions whose names appear in the provided
    collection of reaction names. Every string in the provided collection
    must be the name of a reaction in this fluxome, but the names of internal
    and sink reactions are ignored.

    :   **Specified by:**: `getSourceRxns` in interface `IrrevRxnFluxome`
    :   **Parameters:**: `rxnNames` - a collection of reaction names. **Returns:**: the names of source reactions contained in `rxnNames` mapped to their corresponding reaction. **Throws:**: `java.lang.IllegalArgumentException` - if a string in `rxnNames` is not the name of a reaction in this fluxome.

---


### isReqCatalyst

```
public boolean isReqCatalyst(java.lang.String catalyst,
                             java.lang.String rxnName)
```

:   Indicates if the specified catalyst is required for the reaction with
    the specified name.

    :   **Specified by:**: `isReqCatalyst` in interface `CatalyzedFluxome`
    :   **Parameters:**: `catalyst` - the catalyst.: `rxnName` - the name of the reaction. **Returns:**: `true` if the provided catalyst is required by the reaction with the provided name; `false` otherwise. **Throws:**: `java.lang.IllegalArgumentException` - if `rxnName` is not a valid reaction name.

---


### areReqCatalysts

```
public boolean areReqCatalysts(java.util.Collection<java.lang.String> catalysts,
                               java.lang.String rxnName)
```

:   Indicates if the provided collection of catalysts is required for the
    reaction with the specified name.

    :   **Specified by:**: `areReqCatalysts` in interface `CatalyzedFluxome`
    :   **Parameters:**: `catalysts` - the collection of catalysts.: `rxnName` - the name of the reaction. **Returns:**: `true` if the provided catalysts are required by the reaction with the provided name; `false` otherwise. **Throws:**: `java.lang.IllegalArgumentException` - if `rxnName` is not a valid reaction name.


---


|  |  |  |
| --- | --- | --- |
| **Overview** \| **Package** \| **Class** \| **Use** \| **Tree** \| **Deprecated** \| **Index** \| **Help** | |  |
| **PREV CLASS**   **NEXT CLASS** | **FRAMES**    **NO FRAMES**     **All Classes** |  |
| SUMMARY: NESTED \| FIELD \| CONSTR \| METHOD | DETAIL: FIELD \| CONSTR \| METHOD |  |


---
